# Consumption of Alcoholic Stimulants in Hospitals—Gairdner

**Published:** 1861-08

**Authors:** 


THE LAUDABLE object and example of Dr. Gairdner in publishing the statistics of the consumption of alcoholic stimulants by the hospital patients under his charge, should certainly stimulate all hospital physicians to prepare accurate records of the actual expenditure of alcoholic stimulants. In how many various ways this would lead to extremely valuable results, scientifically, morally, economically, as well as therapeutically, we need not here point out. We quote table of

*Average Daily Consumption of Alcoholic Stimulants, per Patient, during Five Successive Years, in the Royal Infirmary, Wards* 4, 15, *and* 16.

| | 1856. | 1857. | 1858. | 1859. | 1860. |
|---|---|---|---|---|---|
| GENERAL WARD—Males— | | | | | |
| Wines, (ounces), | 0.158 | 0.465 | 0.710 | 0.928 | 0.739 |
| Spirits, (ounces), | 0.056 | 0.312 | 0.287 | 0.184 | 0.454 |
| Malt Liquors, (pints), | 0.039 | 0.040 | 0.025 | 0.053 | 0.058 |
| GENERAL WARD—Females— | | | | | |
| Wines, (ounces), | 0.446 | 0.534 | 0.799 | 1.498 | 1.200 |
| Spirits, (ounces), | 0.295 | 0.312 | 0.223 | 0.164 | 0.510 |
| Malt Liquors, (pints), | 0.064 | 0.069 | 0.048 | 0.061 | 0.048 |
| FEVER WARD—Females— | | | | | |
| Wines, (ounces), | 0.715 | 1.256 | 1.734 | 1.725 | 1.140 |
| Spirits, (ounces), | 0.069 | 0.083 | 0.346 | 0.052 | 0.135 |
| Malt Liquors, (pints), | 0.023 | 0.029 | 0.135 | 0.069 | 0.027 |

As to the results, Dr. G. avers they are in two particulars most unexpectedly opposed to all his previous impressions. While he fully believed that the use of alcoholic stimulants in his hands had been at least stationary, if not decreasing, the table shows that there has been a nearly uniform increase. Again, it appears that among the women in the general ward the consumption of wine, during the entire period of five years, is decidedly greater than in the corresponding male ward; that of spirits but little less, and that of malt liquors at least as much. As the two wards are very much alike as regards the class of cases admitted, and have been during very nearly the whole period subject to exactly the same administration, it is hardly possible that this fact can be accounted for otherwise than by the apparent need for the exhibition of stimulants, and the demand for them having been actually greater among the women than among the men. Dr. G. adds: "I am quite certain, at least, that there is nothing in the personal convictions of myself or my assistants, to account for the fact of so liberal a comparative expenditure of wine on the female side, inasmuch as all my own prejudices and those of most other people, were assuredly in the opposite direction, viz., to the effect that women ought to require, and in the better class of society do require, a less quantity of alcoholic stimulation than men, when suffering under disease, and especially acute disease."

Many other points of Dr. Gs'. article we would willingly quote, but hardly can do them justice. Dr. G. by no means goes the entire length of the late lamented Dr. Todd, and gives stimulants, "if at all, only in very moderate quantities along with the food, and in general, as an aid to the digestion of food; the only exception being in the case of persons largely and habitually dependent upon stimulants from old and formed habits, and in a comparatively small number of acute cases for a very few days, sometimes only a few hours, to help the system over a dangerous crisis, or to co-operate with other needful remedies, such as antimonials, in pulmonary inflammation."

